# Alpha-Ketoglutarate in Low-Protein Diets for Growing Pigs: Effects on Cecal Microbial Communities and Parameters of Microbial Metabolism

**DOI:** 10.3389/fmicb.2018.01057

**Published:** 2018-05-31

**Authors:** Jiashun Chen, Baoju Kang, Qian Jiang, Mengmeng Han, Yurong Zhao, Lina Long, Chenxing Fu, Kang Yao

**Affiliations:** ^1^College of Animal Science and Technology, Hunan Agricultural University, Changsha, China; ^2^Key Laboratory of Agro-Ecological Processes in Subtropical Region, Hunan Provincial Engineering Research Center of Healthy Livestock, Scientific Observing and Experimental Station of Animal Nutrition and Feed Science in South-Central, Ministry of Agriculture, Institute of Subtropical Agriculture, Chinese Academy of Sciences, Changsha, China; ^3^Hunan Collaborative Innovation Center for Utilization of Botanical Functional Ingredients and Hunan Collaborative Innovation Center of Animal Production Safety, Changsha, China; ^4^College of Animal Science and Technology, Northwest A&F University, Yangling, China

**Keywords:** alpha-ketoglutarate, low-protein diet, cecal, bacterial communities, metabolites, growing pigs

## Abstract

Alpha-ketoglutarate (AKG), a critical molecule in the tricarboxylic acid cycle, is beneficial to intestinal functions. However, its influence on intestinal microbiota and metabolism is not fully understood. We investigated the effects of a low-protein (LP) diet supplemented with AKG on cecal microbial communities and the parameters of microbial metabolism in growing pigs. Twenty-seven young pigs (Large White × Landrace) with an average initial body weight of 11.96 ± 0.18 kg were randomly allotted into three groups (*n* = 9): a normal protein (NP) diet containing 20% crude protein (CP); LP diet formulated with 17% CP (LP diet); or LP diet supplemented with 10 g kg^-1^ of AKG (ALP diet). After a 35-day trial period, the digesta of the cecum were collected to analyze the concentrations of ammonia and short-chain fatty acids (SCFAs). We also performed a microbial analysis. Although no significant differences were found in performance among the diet groups, pigs fed the ALP diet had greater average daily gain (ADG) when compared with those in the LP group. Experimental diet did not affect cecal bacterial richness or diversity, as determined by Chao1 and ACE species richness measures and Shannon and Simpson indices, respectively. The predominant phyla Firmicutes, Bacteroidetes, and Proteobacteria increased in relative abundances in the cecum of pigs fed ALP diet. At the genus level, compared to the LP diet, the ALP diet significantly increased the abundances of *Lachnospiraceae* UCG-005, *Lachnospiraceae* NK4A136 group, *Phascolarctobacterium* and *Parabacteroides*, while decreased *Vibrio* and *Maritalea*. Pigs fed the ALP diet increased *Oribacterium* and *Lachnoclostridium* when compared with the NP diet. Non-metric multidimensional scaling analysis revealed that the distribution of microbiota at each group was distinctly clustered separately along principal coordinate. In addition, quantitative PCR revealed that the ALP diet was also associated with increases in the amounts of *Bacteroides, Bifidobacterium*, and *Lactobacillus*, but a decrease in the level of *Escherichia coli*. Compared with the NP diet, the ALP diet enhanced the concentrations of valerate and propionate. This ALP diet also increased the concentrations of valerate and isobutyrate when compared with the LP diet. Moreover, the ALP diet was linked with a significant decline in the concentration of ammonia in the cecum. These results indicate that a LP diet supplemented with AKG can alter the balance in microbial communities, increasing the population of SCFA-producing bacteria and the amounts of *Bacteroides* and *Bifidobacterium*, while reducing the counts of *Escherichia coli* and the amount of ammonia in the cecum.

## Introduction

The gut microbiota play a critical role in regulating and controlling of host energy metabolism (Cani and Delzenne, 2009; Yan et al., 2012). The hindgut of an animal harbors a dense and metabolically active microbiota comprising primarily bacteria that have a profound influence on immunological, nutritional, and physiological processes in the host (Su et al., 2008). Various factors can influence on the composition of the gut microbiota, such as nutritional and non-nutritional dietary components, the intestinal environment, and antibiotic use (Penders et al., 2006). In mammals, proteins and their amino acid-derived metabolites can affect the interactions between the gut microbiota and metabolism (Metges, 2000). Undigested protein in the large intestine can be fermented by indigenous bacteria to form amino acid-derived metabolites (Nyangale et al., 2015; Zhang et al., 2016), such as ammonia (NH_3_), branched chain fatty acids (BCFAs), hydrogen sulfide, and indolic and phenolic compounds, all of which can possibly have toxic impacts on the intestinal epithelium (Geypens et al., 1997; Luo et al., 2015). In pigs, the level of protein in the diet affect the composition and metabolism of bacteria, furthermore, the crystalline amino acids (AAs) -supplemented low-protein (LP) diet may decrease the formation of protein fermentation products, and alter microbial communities in the large intestine (Zhou et al., 2015; Zhang et al., 2016). Moreover, the addition of 3% benzoic acid to a low protein diet can reduce the NH_3_ concentration and NH_3_ emissions by 55 and 57%, respectively (Hansen et al., 2007). Benzoic acid also diminishes the growth of micro-organisms in the intestinal digesta of piglets (Kluge et al., 2006). Therefore, it is important that both researchers and swine managers understand how dietary interventions can lead to differential effects on microbial composition and fermentation.

Alpha-ketoglutarate (AKG) is a central molecule in the tricarboxylic acid cycle (Krebs cycle). It can be rapidly transaminated, by glutamate (Glu) dehydrogenase, to glutamic acid and then further aminated, by glutamine (Gln) synthetase, to form Gln (Prandini et al., 2012; Wang et al., 2016). This conversion is well-established (Wang et al., 2015):

$$\begin{array}{c} {AKG + NH}_{4}{}^{+}+ NADH\to {Glu + NAD}^{+}{+ H}_{2}O\\ {Glu + NH}_{4}{}^{+}+ ATP\to Gln + ADP + Pi \end{array}$$

In addition to functioning as both an energy donor and a scavenger of ammonium ions, AKG can provide a source of Gln that stimulates protein synthesis in intestinal epithelial cells (Yao et al., 2012). Furthermore, Junghans et al. (2006) reported that administration of AKG may markedly affect energy expenditure and nutrient utilization in growing pigs. Dietary supplementation with 1% AKG beneficially modulates the AMPK signaling pathway to improve energy status in the small intestine of LPS-challenged piglets (Hou et al., 2011b). Daily weight gain and feed-efficiency was improved when pigs were fed supplemented with AKG (Hou et al., 2011a; Chen et al., 2017a). In addition, the *in vitro* bacterial community structure is changed when AKG is introduced into diluted rumen fluid (Zhang et al., 2011). We have previously shown that dietary supplementation with AKG can potentially promote the growth of beneficial bacteria, improve intestinal microbial populations, modulate the production of short-chain fatty acids (SCFAs), and decrease the level of NH_3_ in the gut of growing pigs (Chen et al., 2017b).

Our study objective was to investigate how combining a LP diet with AKG might influence growth performance, cecal microbial communities, and parameters of microbial metabolism in young pigs. The data obtained here will help us further understand the mechanism through which AKG improves pig performance, and provide a scientific basis for using AKG in commercial practice.

## Materials and Methods

These experiments were conducted in accordance with Chinese guidelines for animal welfare and experimental protocols, and all procedures were approved by the Committee of Animal Care at the Institute of Subtropical Agriculture, Chinese Academy of Sciences. The AKG component (purity ≥ 98%) was obtained from Hubei Yuancheng Saichuang Technology Co., Ltd., Wuhan 430064, China.

### Animals, Housing, and Experimental Treatments

Twenty-seven growing pigs (Large White × Landrace, 44 ± 1 day of age) with an average initial body weight of 11.96 ± 0.18 kg were randomly assigned to one of three dietary treatments (*n* = 9). The pigs were housed individually in cages (1.5 m × 1.5 m). Their experimental diets were formulated, based on corn–soya bean meal, to be isoenergetic and meet the nutritional needs of these animals according to the National Research Council (NRC, 2012) (**Table 1**). The following dietary treatments were used: (1) NP, a normal recommended protein diet containing approximately 20% CP; (2) LP, a LP diet formulated to contain approximately 17% CP (3 percentage points below the recommended level); and (3) ALP, a LP diet supplemented with 10 g kg^-1^ AKG as a substitute for the regular corn components. The experiment lasted 35 days, during which time all of the pigs had *ad libitum* access to clean drinking water and their assigned diets.

**Table 1:** Ingredient composition and nutrient levels in experimental diets (as-fed basis, %).

Item	Dietary treatment^a^		
	NP	LP	ALP
**Ingredient (%)**			
Corn	63.64	66.50	65.50
Soybean meal	19.80	18.80	18.80
Dried whey	4.30	4.30	4.30
Fish meal	9.00	4.00	4.00
Soybean oil	0.80	2.60	2.60
AKG^b^	0.00	0.00	1.00
Limestone	0.50	0.60	0.60
Monocalcium phosphate	0.00	0.74	0.74
L-lysine-HCl	0.41	0.65	0.65
L-threonine	0.11	0.25	0.25
DL-methionine	0.13	0.20	0.20
L-tryptophan	0.01	0.06	0.06
Sodium chloride	0.30	0.30	0.30
Premix^c^	1.00	1.00	1.00
Total	100.00	100.00	100.00
**Nutrient level (%)**			
Digestible energy (MJ/kg)^d^	14.60	14.60	14.60
Crude protein	20.19	17.21	17.20
Lysine	1.25	1.24	1.23
Methionine	0.38	0.37	0.37
Methionine + cysteine	0.62	0.65	0.63
Threonine	0.76	0.73	0.74
Tryptophan	0.22	0.22	0.21
Total calcium	0.72	0.72	0.71
Total phosphorus	0.65	0.64	0.63

*^*a*^NP, normal protein; LP, low protein; ALP, AKG plus low protein. ^*b*^AKG, alpha-ketoglutarate. ^*c*^Supplied per kg of diet: CuSO_4_⋅5H_2_O, 19.8 mg; KI, 0.20 mg; FeSO_4_⋅7H_2_O,400 mg; NaSeO_3_, 0.56 mg; ZnSO_4_⋅7H_2_O, 359 mg; MnSO_4_⋅H_2_O, 10.2 mg; Vitamin K (menadione), 5 mg; Vitamin B_1_, 2 mg; Vitamin B_2_, 15 mg; Vitamin B_12_, 30 g; Vitamin A, 5400 IU; Vitamin D_3_, 110 IU; Vitamin E, 18 IU; Choline chloride, 80 mg; Antioxidants, 20 mg; Fungicide, 100 mg. ^*d*^Calculated values, other values were measured directly.*

### Sample Collection and Preparation

Body weights and feed consumption were measured at the beginning and end of the 35-day experiment period. Those data were used for calculating average daily gain (ADG), average daily feed intake (ADFI), and the feed-to-gain ratio (F/G). When the feeding test ended, all of the animals were immediately anesthetized with sodium pentobarbital (50 mg/kg body weight) and killed by jugular puncture and eviscerated (He et al., 2009). Afterward, the luminal digesta of the cecum were collected and stored at -80°C before determining the concentrations of SCFAs and ammonia nitrogen (NH_3_–N), as well as the composition and diversity of the gut microbiota.

### Analysis of Short-Chain Fatty Acids and Ammonia Nitrogen in the Cecal Digesta

The digesta of cecum were homogenized and centrifuged at 1000 × *g* and 4°C for 10 min. Afterward, a mixture of supernatant fluid and 25% metaphosphoric acid solution (4 mL:1 mL) was prepared for the determination of SCFAs (i.e., acetic, propionic, butyric, and valeric acids) and BCFAs (i.e., isobutyric and isovaleric acids). Analysis via gas chromatography was conducted as previously described (Kong et al., 2009).

The concentrations of NH_3_–N were calculated according to Nessler’s reagent colorimetric method, as described by Diao et al. (2014). Briefly, the supernatants of the cecal digesta samples were centrifuged at 5000 × *g* for 15 min after adding 1:10 ammonia-free water. Afterward, 1 mL of the supernatant was transferred to a 50 mL sterile tube to which 49 mL of ammonia-free water and 1 mL of potassium sodium tartrate solution were added before the mixture was briefly blended with an onavortex mixer. After 1.5 mL of Nessler’s reagent was added, the mixture stood for 10 min. Absorbance was read at 420 nm against ammonia-free water on a UV-vis Spectrophotometer (UV1100; MAPADA, Shanghai, China). Based on the absorbance of the standard, we calculated the NH_3_–N concentrations as follows: NH_3_–N (N, mg/L) = *m* × 1000*/V*, where *m* represented the NH_3_–N concentration corrected by the standard curve and *V* represented the volume of the supernatant.

### Bacterial Quantification by Real-Time PCR

Total bacterial DNAs were extracted from the contents of each intestinal sample (0.2 g) according to a previously described protocol (Kraler et al., 2016), using a commercially available QIAamp DNA Stool Mini Kit (Qiagen, Hilden, Germany). Those extracts were stored at -80°C. They were then quantified on a Nanodrop 2000 Spectrophotometer (Thermo Scientific, Courtaboeuf, France) before the results were adjusted to a concentration of 10 ng μL^-1^.

Methods based on 16S rRNA were used to assess the abundances of total bacteria, *Bacteroides, Bifidobacterium, Firmicutes, Escherichia coli, Lactobacillus, Clostridium coccoides*, and the *Clostridium leptum* subgroup, as described previously (Kong et al., 2014; Fleury et al., 2016). All PCR primers used in this study are listed in **Table 2**. Duplicate sample analysis was performed in a mixture (final volume, 10 μL) that contained 1 μL of diluted DNA sample, 0.2 μM of each primer, and 0.25 μM of TaqMan probe, using a 1× of SYBR Premix Ex Taq II kit (TaKaRa Bio Inc., Shiga, Japan). The qPCR protocol for assaying *E. coli* included 0.3 μM of each primer and 0.1 μM of probe, while those reactions for *Firmicutes, Clostridium coccoides*, and the *Clostridium leptum* subgroup used a concentration of 0.4 μM of each primer. The amplification program entailed 95°C for 30 s; followed by 40 cycles of 95°C for 5 s and 60°C for 30 s; and then a final melting-curve for SYBR Green tests. The melting curve analysis and size-determination of amplificates on agarose gels verified that the target fragments had been amplified. Standard curves were generated as described by Qi et al. (2011). For each sample and each bacterial group, results were expressed in log10 copies of 16S rRNA genes per g of intestinal content material (Metzlerzebeli et al., 2015).

**Table 2:** Sequences of primers and probes used for group-specific quantitative PCR.

Bacterial group/species		Sequences of primers and probes (5′to 3′)	References
Total bacteria	Forward	CGG TGA ATA CGT TCC CGG	Fleury et al., 2016
	Reverse	TAC GGC TAC CTT GTT ACG ACT T	
	Probe	(6FAM)CTTGTACACACCGCCCGTC(BHQ1)	
*Bacteroides*	Forward	CCT WCG ATG GAT AGG GGT T	Fleury et al., 2016
	Reverse	CAC GCT ACT TGG CTG GTT CAG	
	Probe	(6FAM)AAGGTCCCCCACATTG(BHQ1)	
*Bifidobacterium*	Forward	CGG GTG AGT AAT GCG TGA CC	Fleury et al., 2016
	Reverse	TGA TAG GAC GCG ACC CCA	
	Probe	(6FAM)CTCCTGGAAACGGGTG(BHQ1)	
*Escherichia coli*	Forward	CAT GCC GCG TGT ATG AAG AA	Fleury et al., 2016
	Reverse	CGG GTA ACG TCA ATG AGC AAA	
	Probe	(6FAM)TATTAACTTTACTCCCTTCCTCCCCGCTGA(BHQ1)	
*Lactobacillus*	Forward	AGC AGT AGG GAA TCT TCCA	Fleury et al., 2016
	Reverse	CGC CAC TGG TGT TCY TCC ATA TA	
*Firmicutes*	Forward	GTCAGCTCGTGTCGTGA	Kong et al., 2014
	Reverse	CCATTGTATACGTGTGT	
*Clostridium leptum* subgroup	Forward	GCACAAGCAGTGGAGT	Kong et al., 2014
	Reverse	CTTCCTCCGTTTTGTCAA	
*Clostridium coccoides*	Forward	CGGTACCTGACTAAGAAGC	Kong et al., 2014
	Reverse	AGTTTTATTCTTGCGAACG	

### DNA Extraction, PCR Amplification, Library Preparation and Sequencing

Fifteen growing pigs from the three treatment groups (5 per group) were randomly chosen for 16S rRNA sequencing analyses. Total genomic DNA was isolated from the samples of cecal digesta by using QIAamp DNA Stool Mini Kits according to the manufacturer’s instructions. The concentration of the extracted DNA was determined with the NanoDrop-1000 Spectrophotometer (NanoDrop Technologies Inc., Wilmington, DE, United States), and was stored at -80°C before further analysis.

To analyze the taxonomic composition of the bacterial community, we used 16S rRNA gene amplicons to determine the diversity and make structure comparisons of the bacterial communities in each of these samples. Sequencing was performed at Novogene Bioinformatics Technology Co. Ltd., Beijing, China. The PCR amplifications were conducted with the barcoded primer pair 341f/806r set, which amplifies the V3–V4 fragments of the 16S rDNA gene (341F:CCTAYGGGRBGCASCAG, 806R: GGACTACNNGGGTATCTAAT) (Muyzer et al., 1993; Caporaso et al., 2011). PCR reactions were performed in a volume of 30 μL containing 12 μL sterile water, 1.0 μL DNA template, 1.0 μL of each primer, and 15 μL 2 × Phusion Master Mix (New England Biolabs, United States). The PCR cycle conditions were as follows: initial denaturation at 98°C for 1 min, followed by 30 cycles at 98°C for 10 s, 50°C for 30 s, and 72°C for 30 s, and a final extension step at 72°C for 5 min. Resulting amplicons were confirmed on 2% agarose gels containing ethidium bromide.

All amplicons were in the size range of 400–450 bp, and were purified using a GeneJET Gel Extraction Kit (Thermo Fisher Scientific, Carlsbad, CA, United States). Following quantitation, equal concentrations of the purified amplicons were combined into a single tube. Sequencing libraries were generated using a NEB Next Ultra DNA Library Prep Kit for Illumina (New England Biolabs, Ipswich, MA, United States) following manufacturer’s recommendations, and index codes were added. The library quality was assessed on a Qubit@ 2.0 Fluorometer (Thermo Fisher Scientific, Carlsbad, CA, United States) and Agilent Bioanalyzer 2100 system. Sequencing was conducted on an Illumina MiSeq2500 platform, which generated 250-bp paired-end reads.

### Bioinformatics Analysis

Raw data were screened and assembled by FLASH v1.2.7^1^ (Magoč and Salzberg, 2011) and QIIME (V1.7.0)^2^ (Caporaso et al., 2010) software packages. Chimera sequences were removed through the UCHIME algorithm (UCHIME Algorithm^3^) (Edgar et al., 2011) to form the “effective sequences” collection for each sample. Uparse software (Uparse v7.0.1001)^4^ (Edgar, 2013) were used for analysis of the sequences and determination of the Operational Taxonomic Units (OTUs) with an identity threshold of 97%. Meanwhile, we picked a representative sequence for each OTUs and used the RDP classified to assign taxonomic data to each representative sequence. Taxonomy classifications were assigned against RDP Classifier (Version 2.2)^5^ (Wang et al., 2007) and the Greengenes database^6^ (DeSantis et al., 2006). The taxon abundance of each sample was then determined at the levels of phylum, class, order, family, and genera. All of the analyses from clustering to alpha diversity (Chao1, ACE, Simpson and Shannon) were calculated by QIIME software (Version 1.7.0) and displayed with R software (Version 2.15.3). Non-metric Multidimensional Scaling (NMDS) plots based on Bray–Curtis distance was performed to show the group differences in microbial community structure. Statistical testing among variation in microbial community composition was carried out using the analysis of similarity (ANOSIM). Hierarchical clustering of samples was completed using UPGMA. All the raw sequence data in the present study have been deposited in the sequence read archive (SRA) at NCBI under the accession number SRP125897.

### Statistical Analysis

Growth performance indices were assessed by analysis of variance (ANOVA). A non-parametric ANOVA (Kruskal–Wallis test) was performed to analyze cecal SCFAs, ammonia concentrations and bacterial populations by qPCR. Differences in alpha diversity and relative abundances of bacterial phyla between samples were analyzed by the Mann–Whitney *U*-test. All analyses were performed using IBM SPSS v. 20.0 (SPSS Inc., Chicago, IL, United States). For all tests, values of *P* < 0.05 were considered statistically significant differences. The abundance of genus with significant differences between groups were assessed by analysis of MetaStat, significant correlations were found (*q* < 0.05), extremely significant differences was considered at *q* < 0.01.

## Results

### Growth Performance

**FIGURE 1:**
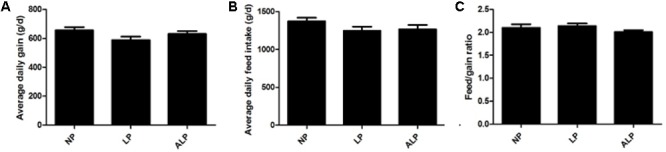
The growth performance of growing pigs fed various experimental diets. **(A)** Average daily gain of LP-diet-fed growing pigs with AKG supplementation. **(B)** Average daily feed intake of LP- diet-fed growing pigs with AKG supplementation. **(C)** Feed intake/gain of LP-diet-fed growing pigs with AKG supplementation. ADG, average daily gain; ADFI, average daily feed intake; F/G, feed intake/gain. NP, normal protein; LP, low protein; ALP, AKG plus low protein. Data are presented as the means ± SEM (*n* = 9). Differences were assessed by ANOVA.

The effects of LP diets supplemented with AKG on feed intake and growth performance are shown in **Figure 1**. Among the diet groups, no significant differences were found in ADG (*P* = 0.099), ADFI (*P* = 0.282), or F/G (*P* = 0.326). However, pigs fed the ALP diet showed a 7.23% increase in average daily gain when compared with those in the LP group (*P* = 0.148).

### DNA Sequence Coverage and Microbial Community Structure in Cecal Digesta

To evaluate the impact of a LP diet supplemented with AKG on microbial communities, a total number of 1,055,010 of V3–V4 16S rRNA valid sequences reads were obtained from 15 samples (70,334 ± 1,000 per sample), including an average of 66,241 (NP group), 72,001 (LP), and 72,760 (ALP) raw reads in the current study. After trimming, assembly, and quality filtering, an average of 35,384, 39,546, and 41,270 sequences were selected for further analysis from the NP, LP, and ALP groups, respectively. When all of the samples were considered, the sequence reads numbered 32,694–45,537 per sample, with an average of 38,384 (Supplementary Table S1). A total of 30,000 effective sequences was extracted from each sample for comparisons at the same sequencing depth.

**FIGURE 2:**
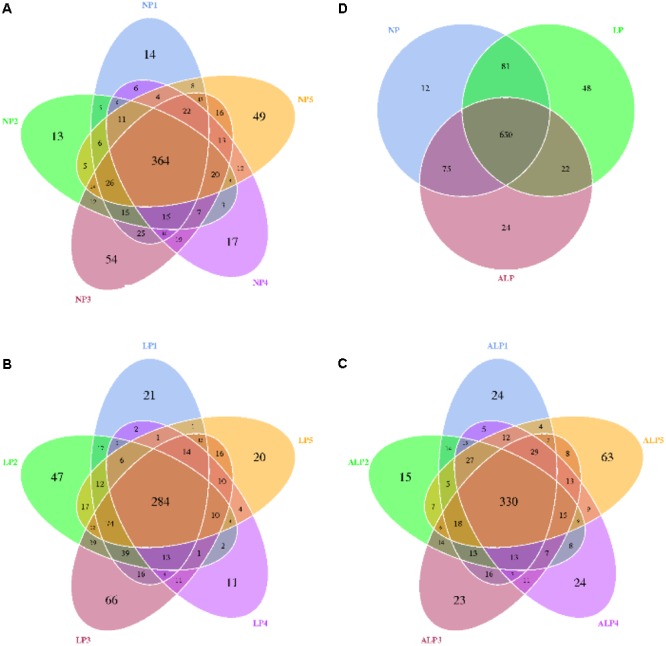
Shared OTUs analysis of the different samples. Venn diagram showing the unique and shared OTUs in the different samples. NP, LP, and ALP are samples mentioned in Supplementary Table S1. **(A)** for NP1, NP 2, NP 3, NP 4, and NP 5 samples; **(B)** for LP1, LP 2, LP 3, LP 4, and LP 5 samples; **(C)** for ALP1, ALP 2, ALP 3, ALP 4, and ALP 5 samples; **(D)** for NP, LP, and ALP samples; NP, normal protein; LP, low protein; ALP, AKG plus low protein.

**FIGURE 3:**
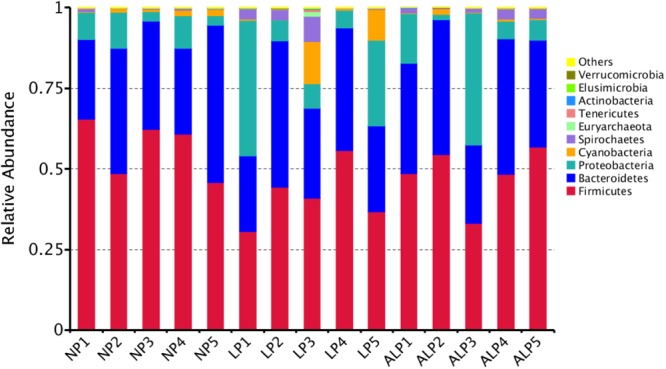
Relative abundance of cecal bacteria, at phylum level, in pigs fed different diets. Representative sequences for each OTU were used to annotate taxonomic information. NP, normal protein; LP, low protein; ALP, AKG plus low protein.

**FIGURE 4:**
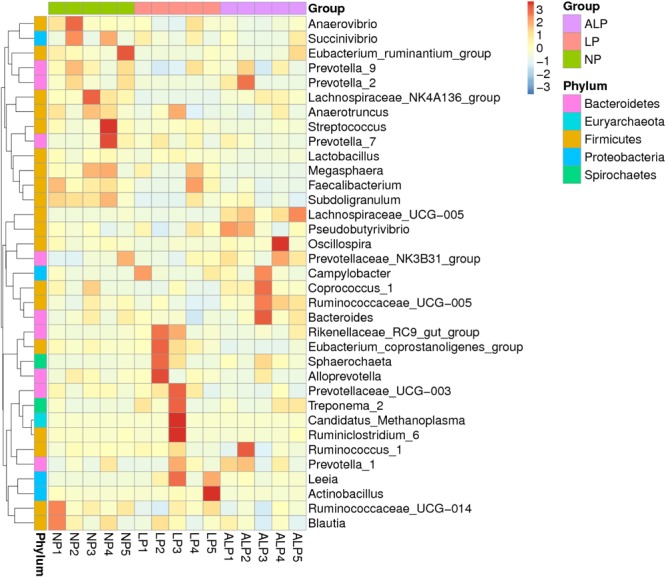
Heatmap based on hierarchical clustering solution (Bray–Curtis distance metric and complete clustering method) for 15 pigs fed three different diets, shown across columns. Rows represent top 35 microbial genera. NP, normal protein; LP, low protein; ALP, AKG plus low protein.

**FIGURE 5:**
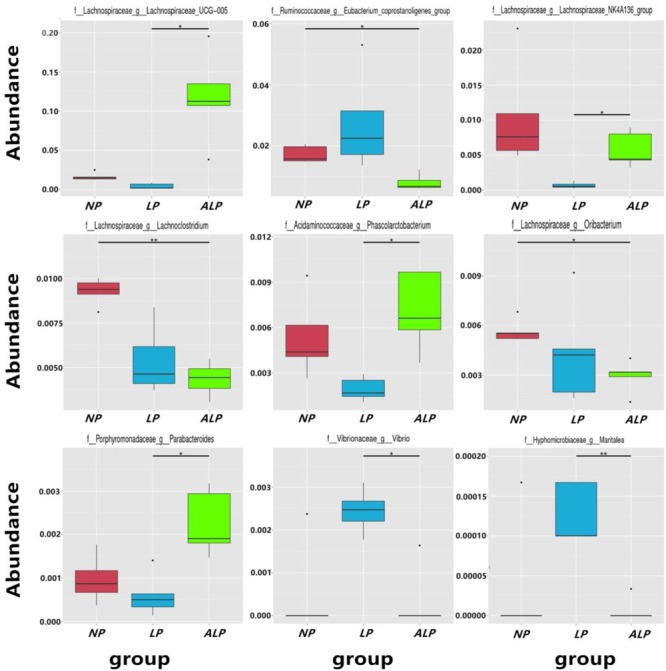
Relative abundance of microbial genera (percentage) in pig cecum that were significantly affected by dietary treatment. NP, normal protein; LP, low protein; ALP, AKG plus low protein. ^∗^Indicates significant difference between the two groups (*q* value < 0.05), ^∗∗^indicates the difference between the two groups was significant (*q* value < 0.01).

A total of 8868 OTUs were obtained after filtering out reads with low quality segments, with an average of 591 ± 14 OTUs per sample, and from the Venn analysis of OTUs (**Figures 2A–C**), 11, 48, and 24 unique OTUs were identified in NP, LP, and ALP groups, respectively(Supplementary Table S1 and **Figure 2D**). At a genetic distance of 3%, no remarkable differences were found in the diversity indices (Shannon and Simpson) and richness estimators (ACE and Chao1) of the cecal microbiota among treatment groups (*P* > 0.05) (Supplementary Figure S1 and Supplementary Table S2). At the phylum level, five phyla – Bacteroidetes, Cyanobacteria, Firmicutes, Proteobacteria, and Spirochaetes – had a relative abundance greater than 0.5% in at least one experimental group. Of these, Firmicutes predominated in all samples, with a relative abundance of 41.69–56.57%, followed by Bacteroidetes (32.25–35.12%), Proteobacteria (7.11–14.04%), Cyanobacteria (0.58–4.78%) and Spirochaetes (0.41–2.83%). When the LP diet was supplemented with AKG, widespread changes were induced in the gut microbial community structure at the phylum level when compared with the LP diet alone. In particular, abundances were increased for Firmicutes (48.23% vs. 41.69%) (*P* = 0.310) but decreased for Cyanobacteria (0.06% vs. 4.78%) (*P* = 0.421) and Spirochaetes (1.80% vs. 2.83%) (*P* = 0.690). In contrast, both Proteobacteria (14.04% vs.7.11%) (*P* = 0.841) and Spirochaetes (1.80% vs.0.41%) (*P* = 0.095) were increased in the ALP group when compared with the NP group (**Figure 3**). The proportion of some phyla (Euryarchaeota, Tenericutes, Actinobacteria, Elusimicrobia, and Verrucomicrobia) was less than 1% of total microbial community. Within the bacterial population, the top 35 genera were identified across all samples (**Figure 4**). The abundance of genus *Lachnospiraceae* UCG-005(11.76% vs. 0.42%) (*q* = 0.041), *Lachnospiraceae* NK4A136 group (0.58% vs. 0.07%) (*q* = 0.042) in the Lachnospiraceae family, *Phascolarctobacterium* (0.71% vs. 0.19%) (*q* = 0.033) in the Acidaminococcaceae family and *Parabacteroides* (0.23% vs. 0.06%) (*q* = 0.012) in the Porphyromonadaceae family were increased in the ALP group when compared with the LP group, while *Vibrio* (0.03% vs. 0.24%) (*q* = 0.032) in the Vibrionaceae family and *Maritalea* (0.00067% vs. 0.01%) (*q* < 0.01) in the Hyphomicrobiaceae family were decreased. Supplementing the LP diet with AKG caused the abundances of *Oribacterium* (0.57% vs. 0.29%) (*q* = 0.021) and *Lachnoclostridium* (0.93% vs.0.44%) (*q* < 0.01) in the Lachnospiraceae family to increase when compared with the NP group (**Figure 5**). Furthermore, the composition of *Oribacterium*, Lachnospiraceae and *Phascolarctobacterium* in cecal samples based on bacterial quantification by real-time PCR (Supplementary Material) was represented in Supplementary Figure S2. The amounts of Lachnospiraceae and *Phascolarctobacterium* were significantly increased in pigs receiving the ALP diets when compared with the LP group (*P* = 0.027). Supplementing the LP diet with AKG caused the abundances of *Oribacterium* (*P* = 0.042) to increase when compared with the NP group.

**FIGURE 6:**
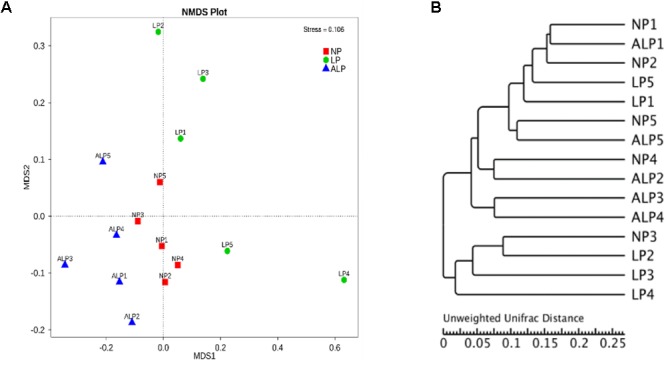
Comparison of cecal microbial community structures within growing pigs fed different diets. **(A)** Non-metric multidimensional scaling (NMDS) based on weighted UniFrac distance among samples of different treatments, based on stress < 0.2 with significantly different abundance among groups (NP, LP, and ALP), **(B)** UPGMA tree, all revealing significant differences among treatments based on Unweighted UniFrac distances of OTU community. NP, normal protein; LP, low protein; ALP, AKG plus low protein.

The relationships in the cecal bacterial communities between samples were compared by NMDS and ANOSIM based on Bray–Curtis (Singh et al., 2015). The NMDS plot indicated that the distribution of microbiota at each group was distinctly clustered separately along principal coordinate (**Figure 6A**). ANOSIM revealed significant differences in the structure of gut microbiota among different groups (**Figure 6A**; ALP-NP *r* = 0.648, *p* = 0.004; LP-NP *r* = 0.168, *p* = 0.043; ALP-LP *r* = 0.204, *p* = 0.078), indicating that ALP diet could exert a notable effect on the cecal microbial community of piglets. Furthermore, the HCA indicated that the similarity dendrogram was separated by AKG rather than protein level (**Figure 6B**).

### Quantitative Analysis of the Intestinal Microbiota

**FIGURE 7:**
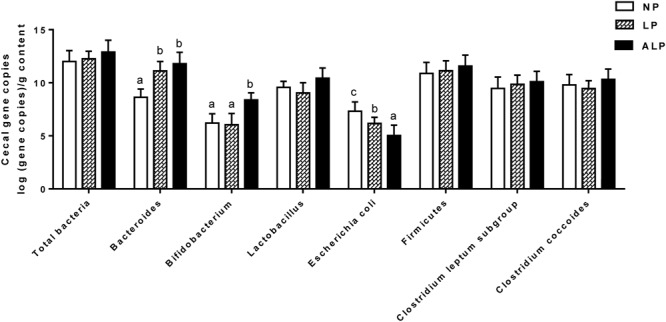
Comparison of major bacterial taxonomic populations in cecum among dietary treatments, using real-time PCR analysis. NP, normal protein; LP, low protein; ALP, AKG plus low protein. For each bacterial group, bars not labeled with same letters indicate values are significantly different at *P* < 0.05.

To examine the quantitative changes to intestinal microbiota in the cecum, we performed real-time PCR for the total bacteria population as well for some major bacterial groups. **Figure 7** shows that the amounts of *Bacteroides* were significantly increased in pigs receiving the ALP and LP diets when compared with levels in pigs fed the NP diet (*P* = 0.032). Moreover, the amounts of *Bifidobacterium* significantly increased (*P* = 0.013), *Escherichia coli* significantly decreased (*P* = 0.025), and the population of *Lactobacillus* slightly increased (*P* = 0.081) in the ALP group when compared with the NP and LP groups. However, no significant differences among treatment groups (*P* > 0.100) were found for any of the other examined populations in the cecum.

### Fermentation Metabolites of Cecal Digesta

**FIGURE 8:**
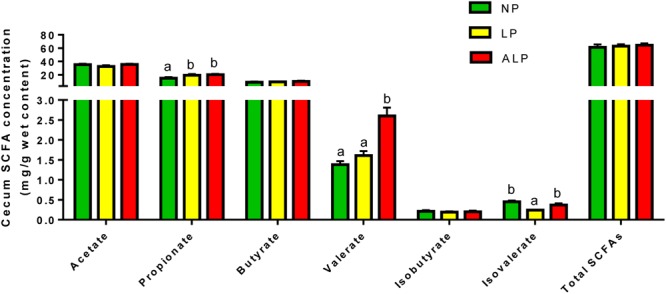
Comparison of cecal short chain fatty acids (SCFAs) (mg/g) among dietary treatments. NP, normal protein; LP, low protein; ALP, AKG plus low protein. For each group of acids, bars not labeled with same letters indicate values are significantly different at *P* < 0.05.

The concentrations of SCFAs and ammonia in cecal digesta are shown in **Figure 8**. While the concentrations of acetate, butyrate, isobutyrate, and total SCFAs did not differ significantly among groups, the isobutyrate concentration was significantly decreased (*P* = 0.021) in response to the LP diet. The valerate concentration was significantly decreased (*P* = 0.019) due to the LP diet when compared with the effect of the ALP diet, while the levels of propionate were significantly increased in animals fed with either the LP or ALP diets (*P* = 0.038) when compared with the NP group.

**FIGURE 9:**
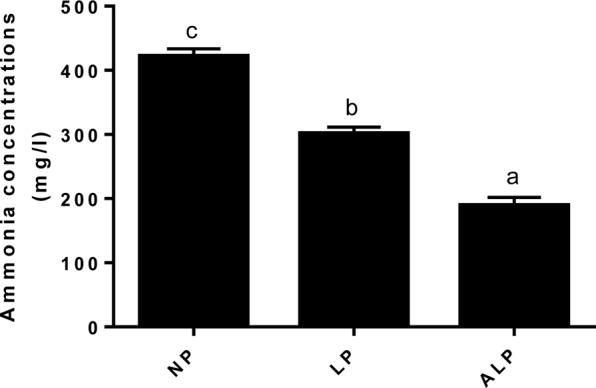
Comparison of ammonia nitrogen (mg/L) concentrations among dietary treatments. NP, normal protein; LP, low protein; ALP, AKG plus low protein. Bars not labeled with same letters indicate values are significantly different at *P* < 0.05.

Ammonia is produced through bacterial deamination of amino acids. Compared with the NP diet, the LP and ALP diets were linked with significant reductions (*P* = 0.002) in the ammonia concentration in the cecum (**Figure 9**).

## Discussion

Organic acids and their salts are considered a class of natural growth promoters that have long been used as feed additives in pig nutrition programs to reduce the buffering capacity of starter diets (Giannenas et al., 2016). Various organic acids (e.g., formic, citric, fumaric, malic, and benzoic) can improve growth performance and regulate the intestinal microbiota in pigs (Partanen and Mroz, 1999; Piva et al., 2002; Kluge et al., 2006; Øverland et al., 2008; Halas et al., 2010). However, the strategy of using a low protein diet balanced with AAs has no influence on growth performance in commercial swine operations (Opapeju et al., 2008) and broiler chickens (Kobayashi et al., 2013). Our research goal was to examine how, while applying current production practices, a low protein diet (3% below normal) could be supplemented with AKG to improve growth performance, the microbial communities, and metabolic profiles in the cecum of a growing pig. In fact, the LP diet alone had no impact on the values calculated for ADG, ADFI, or F/G. This outcome is consistent with that reported by Le Bellego and Noblet (2002), who showed that reducing the protein level from 204 to 169 g/kg did not affect feed intake nor daily weight gain of the piglets (12 kg initial BW). However, we did note here that the ADG was increased by 7.23% in pigs that received an LP diet supplemented with AKG when compared with the results from the LP group. This is in accord with findings from previous studies (Hou et al., 2011a; Chen et al., 2017a). It is possible that the dietary AKG supplementation promotes intestinal development (Hou et al., 2010), and increases AA- and energy-digestibility and nitrogen deposition (Chen et al., 2017a), while reducing the populations of harmful bacteria and modifying their metabolites within the gastrointestinal tract (Chen et al., 2017b). Furthermore, AKG is an intermediate of the citric acid cycle and the natural ubiquitous collector of amino groups in body tissues. It has been reported that AKG can stimulates AMPK phosphorylation and oxidation of energy substrates in the intestinal mucosa (Hou et al., 2011b), thereby inactivating one of its downstream target enzymes, acetyl-CoA carboxylase (ACC) (Dyck et al., 1999), which converts acetyl-CoA into malonyl-CoA (an inhibitor of fatty acid oxidation and a substrate for fatty acid synthesis) (Hou et al., 2011b) and enhancing ATP supply, thereby improving fat storage and weight gain. This was reflected by the rise in ADG in those pigs fed ALP diets over the entire experimental period.

Because the gut microbial community has potentially important roles in swine growth performance and health, its composition and metabolic activities in various nutritional and physiological contexts deserve close attention (Hill et al., 2005). It has been demonstrated that the composition of the intestinal and fecal microbiota (including the bacterial profiles of the mucosa and digesta) changed in pigs from birth to the finishing phases (Konstantinov et al., 2006; Kim et al., 2011). We comprehensively evaluated the diversity in that microbial community as well as the composition of cecal samples from growing pigs by using 16S rRNA gene sequencing. On average, 38,384 effective reads were obtained for each sample (Supplementary Table S1), with high coverage (>99.07%) (Supplementary Table S2). Furthermore, the microbial richness and diversity indices in the cecum were not influenced by diet (Supplementary Figure S1 and Supplementary Table S2). At the phylum level, the cecal microbial communities in growing pigs were dominated by Firmicutes (41.69–56.57% of the total microbial content), Bacteroidetes (32.25–35.12%), and Proteobacteria (7.11–14.04%) (**Figure 3**). This observation is in accordance with the findings of Ji et al. (2017) and Isaacson and Kim (2012). We also found that, at the genus level, the AKG-supplemented LP diet increased the abundances of Lachnospiraceae, and *Phascolarctobacterium* species from the Firmicutes phylum, and *Parabacteroides* species from the Bacteroidetes phylum (**Figures 4, 5**). Some bacteria belonging to the Firmicutes phylum can efficiently ferment fiber into butyrate, while some bacteria from the Bacteroidetes phylum ferment fiber into acetate and propionates (Zhou et al., 2017). Moreover, microbial community structure in piglets fed with ALP was remarkably different from that in LP group or NP (**Figure 6A**). Furthermore, the HCA showed that the similarity dendrogram was separated by AKG rather than protein level (**Figure 6B**). This suggests that the change in gut microbiota structure results from its accommodation to an intestinal environment created by adding AKG to the diet. As described below, there were some changes in intestinal microbial composition, and consequently altered metabolites types and concentrations of cecal digesta under ALP diet.

*Bacteroides* and *Bifidobacterium* are the two main microbial genera that most significantly affect energy homeostasis and substrate fermentation in the hind gut (Tennyson and Friedman, 2008). In pigs, *Bacteroides* species may compose a small fraction of the phylum Bacteroidetes community while other members of this phylum may bound up with the development of obesity (Yan et al., 2012). We found that the LP diet increased the amounts of cecal *Bacteroides* (**Figure 7**), an outcome consistent with that from a previous study (Zhang et al., 2016). The ALP diet also increased the amounts of cecal *Bacteroides* and *Bifidobacterium* (**Figure 7**), perhaps leading to AKG-induced fat accumulations. This was reflected by the rise in ADG in those pigs fed ALP diets over the entire experimental period. These findings partly agree with those described previously. For example, Chen et al. (2017b) demonstrated that supplementing the dietary feed with AKG elevated the cecal *Bifidobacterium* population in growing pigs. Our investigation also revealed that a LP diet supplemented with AKG increased the level of cecal *Lactobacillus* and decreased the *E. coli* population when compared with the other diet groups. These microorganisms – *Lactobacillus* and *E. coli* – are generally considered to be beneficial and harmful, respectively, to the host animal, and *Lactobacillus* is a commonly used probiotic and is thought to create a barrier against pathogen colonization (e.g., by coliform bacteria) in the proximal part of the alimentary tract of young animals (Knarreborg et al., 2002). Because AKG is a weak acid (Filip and Pierzynowski, 2007; Liem et al., 2008), it can only diminish the effect of intestinal pathogenic bacteria (such as coliforms), but not effectively inhibit the activity of useful bacteria, including *Lactobacillus*, largely because of the pH-insusceptibility of that organism (Kazempour and Jahanian, 2017). Furthermore, Glu and several other amino acids are primarily utilized by the bacteria in the intestines of pigs, where they play crucial roles in preserving intestinal functions and health, including the balance of microflora (Dai et al., 2010, 2012).

Profiles of microbial metabolites can be used to represent microbial activity and intestinal health. Some beneficial bacteria (e.g., *Bacteroides* and *Bifidobacterium*) can ferment carbohydrates to produce high concentrations of SCFAs (e.g., acetic acid, butyric acid, valeric acid, and propionic acid), which in turn can decrease the pH of their environment and inhibit the growth of other bacteria (Arunachalam, 1999; Olukosi and Dono, 2014; Chen et al., 2017b). Generally, valeric acids and branched-chain (isobutyric and isovaleric acids) are considered as the end products of protein fermentation (Williams et al., 2015). Protein levels in dietary feed can affect microbial fermentation in the cecum (Zhang et al., 2016). When compared with the NP diet, the LP diet can decrease the amounts of isobutyrate and isovalerate in the cecum (Zhou et al., 2015). Similarly, we found that the LP diet decreased the isobutyrate concentration (**Figure 8**). Furthermore, supplementing this LP diet with AKG increased the concentrations of valerate and propionate (**Figure 8**). We have previously reported that the dietary AKG supplementation enhances the concentrations of butyric acid and valeric acid in the cecal digesta (Chen et al., 2017b). Halas et al. (2010) also showed that dietary benzoic acid supplementation increases the relative amount of valeric acid in the cecum. In the present study, it’s possible that the increase of valerate and propionate could be well explained by the increase of *Bacteroides* and *Bifidobacterium* (Arunachalam, 1999). All of these results suggest that fewer indigestible dietary proteins are entering the cecum and are being degraded by cecal microbial proteases. Energy is the limiting factor for microbial growth in the hindgut, the primary site of intestinal fermentation (Piva et al., 2002). Therefore, AKG can serves as a metabolic fuel for the gut in animals fed an LP diet, thus sparing other oxidative fuels (Lambert et al., 2002). As a valuable source of energy for the intestinal microbiota, AKG improves bacterial fermentation in those parts of the digestive tract, thereby modulating the production of SCFAs and BCFAs.

Ammonia is a toxic catabolite produced through urea hydrolysis and the degradation of microbial amino acids, may interfere with epithelial cell turnover and depress the growth performance of pigs (Walsh et al., 2007). Swine productivity is not necessarily diminished if one can reduce the amount of crude protein and, ultimately, that of ammonia while still maintaining an adequate level of essential amino acids (Canh et al., 1998; Hayes et al., 2004; Hansen et al., 2007). Similarly, we found here that pigs receiving the LP diet showed a decreased ammonia concentration in the cecum (**Figure 9**). That concentration was also significantly reduced in the cecum when the LP diet was supplemented with AKG. These results are in accord with those reported previously (Hansen et al., 2007; Chen et al., 2017b). The LP diet supplementation with crystalline AAs may deter the formation of protein fermentation products in the large intestine (Luo et al., 2015; Zhou et al., 2015). Moreover, the addition of appropriate exogenous organic acids to pig diets may increase the apparent digestibilities of protein and AAs, while also reducing the production of NH_3_ (Partanen and Mroz, 1999; Murphy et al., 2013). All of these findings suggest that deamination and decarboxylation of dietary and/or endogenous protein by microorganisms in the gastrointestinal tract are altered by such supplementation and respond to an LP diet. These responses could be due to a decline in the microbial fermentation. LP diet may decrease amino acid breakdown by cecum bacteria or increased incorporation of amino acids into microbial biomass (Pieper et al., 2012; Zhang et al., 2016). The improvement on amino acids digestion and absorption results in better N digestibility and less NH3 excretion (Chen et al., 2017a). As an intermediate of the citric acid cycle and a natural, ubiquitous collector of amino groups in body tissues, AKG can decrease the level of free NH_3_ by reducing glutamate and glutamine oxidation and/or by incorporating NH_3_ into glutamate by the reversible enzyme GDH (Wirén et al., 2002; Lambert et al., 2006). In addition, dietary AKG supplementation can increase glutamine synthetase gene expression in intestine of common carp (*Cyprinus carpio*) (Dong et al., 2014). Therefore, this supplement has a beneficial effect on N metabolism (Wirén et al., 2002) and can decrease the toxicity of ammonium ions in an organism (Stoll et al., 1991; Velvizhi et al., 2002). Our results confirm those previously showing that AKG is able to replace dietary dispensable AAs by shunting ammonium back into the dispensable AA pool (Filip and Pierzynowski, 2007; Chen et al., 2017b). Moreover, it has been suggested that N retention is good in infants with a bifidus microflora, *Bifidobacterium* promotes the amino acids metabolism. The ability of *Bifidobacterium* to utilize NH_3_ as a source of N may also lead to decrease of NH_3_ in the colon (Arunachalam, 1999). Therefore, we speculate that the decreasing of NH_3_ could be also associated with increasing proportions of *Bifidobacterium* in the current study.

## Conclusion

We investigated the effects on growth performance, cecal microbial communities, and parameters of microbial metabolism in the cecum when pigs were fed a LP diet supplemented with AKG. In particular, this addition altered microbial communities by increasing of the SCFA-producing bacteria and the amounts of *Bacteroides* and *Bifidobacterium*, while also decreasing *Escherichia coli* and the concentration of ammonia in those tissues, thereby increasing ADG of growing pigs. These findings may help us understand the mechanisms by which AKG improves pig performance, and provide additional information that might support the future use of this supplement in animal production.

## Author Contributions

JC and KY conceived the experiment and revised the manuscript. JC, QJ, MH, and BK performed the experiments. JC, QJ, CF, LL, and BK provided the reagents and experimental materials. JC analyzed the data and wrote the manuscript. LL, YZ, and CF prepared the figures and edited the manuscript. All authors reviewed the manuscript.

## Conflict of Interest Statement

The authors declare that the research was conducted in the absence of any commercial or financial relationships that could be construed as a potential conflict of interest.

## Footnotes

1 http://ccb.jhu.edu/software/FLASH/

2 http://qiime.org/index.html

3 http://www.drive5.com/usearch/manual/uchime_algo.html

4 http://drive5.com/uparse/

5 http://sourceforge.net/projects/rdp-classifier/

6 http://greengenes.lbl.gov
