# Supplementary material for: Alpha-Ketoglutarate in Low-Protein Diets for Growing Pigs: Effects on Cecal Microbial Communities and Parameters of Microbial Metabolism
Source: Front Microbiol. 2018 May 31;9:1057. doi: 10.3389/fmicb.2018.01057 (PMC5991137; doi:10.3389/fmicb.2018.01057)
Supplement: Supplementary file 1 [file Presentation_1.pdf]

Supplementary Information

Supplementary table S1. Raw reads and selected effective sequences in each group.

SampleName	Raw PE(#)	Raw Tags(#)	CleanTags(#)	Effective Tags(#)	AvgLen(nt)	Taxon_Tag	OTUs
NP1	67,153	55,811	37,257	34,626	412	31419	607
NP2	68,137	59,236	39,403	37,781	416	35268	580
NP3	64,156	55,752	35,002	32,694	415	29912	655
NP4	63,101	56,062	36,631	34,872	414	33013	585
NP5	68,658	59,725	38,476	36,945	416	33765	641
LP1	74,663	64,716	45,787	44,785	412	43308	592
LP2	71,283	57,286	35,240	34,233	419	31675	634
LP3	73,651	61,828	40,435	39,514	414	37060	668
LP4	67,718	57,163	37,013	35,946	417	34752	430
LP5	72,689	58,262	39,255	38,027	413	36065	554
ALP1	74,005	65,185	46,182	43,014	413	38114	583
ALP2	71,465	63,369	42,723	38,014	414	33612	546
ALP3	76,486	67,685	48,061	45,537	411	42390	589
ALP4	69,260	60,498	40,765	38,408	415	35096	573
ALP5	72,585	63,767	44,355	41,375	413	38223	631

NP, normal protein; LP, low protein; ALP, AKG plus low protein

Supplementary table S2. Number of observed species, richness and diversity indices in the caecal samples from each dietary treatment.

SamplesName	Observed_species	Shannon	Simpson	Chao1	ACE	Good's coverage
NP1	560	6.71	0.97	576.80	584.64	0.998
NP2	526	6.08	0.96	558.04	568.32	0.998
NP3	655	6.88	0.98	736.74	764.03	0.996
NP4	539	6.50	0.97	577.79	576.79	0.998
NP5	591	6.66	0.97	640.09	640.05	0.997
LP1	521	5.11	0.86	561.51	572.03	0.997
LP2	589	6.37	0.96	617.49	622.73	0.998
LP3	634	7.09	0.98	669.18	679.36	0.997
LP4	379	5.52	0.95	414.00	412.99	0.998
LP5	510	5.93	0.95	601.20	583.54	0.997
ALP1	539	6.01	0.95	590.77	589.12	0.997
ALP2	514	6.00	0.95	546.51	556.15	0.998
ALP3	532	4.85	0.82	590.68	609.49	0.997
ALP4	532	6.32	0.96	566.17	564.72	0.998
ALP5	564	6.16	0.95	596.01	600.99	0.998

Supplementary table S3. Sequences of primers and probes used for group-specific quantitative PCR.

Bacterial group/species		Sequences of primers and probes (5′ to 3′)	Sources
Oribacterium	Forward	GCGGCGTGCCTAACACATGC	GenBank: HM120211.1
	Reverse	TTCACCCCAGTCATCAGTCCTGC	
Phascolarctobacterium	Forward	GGC GGC TTA ATA AGT CGA GC	Wu et al., 2017 [10]
	Reverse	CGT TCG CTA CCC TGG CTT TC	
	Forward	TTC GCAAGA ATG AAA CTC AAA	
Lachnospiraceae	Reverse	AAG GAAAGA TCC GGT TAA GGA TC	Newton et al., 2011[11]
	Probe	(6-FAM)-ACC AAG TCT TGA CAT CCG-(MGB)	

Supplementary Figure S1.

Difference analysis of alpha diversity index among NP, LP and ALP groups.

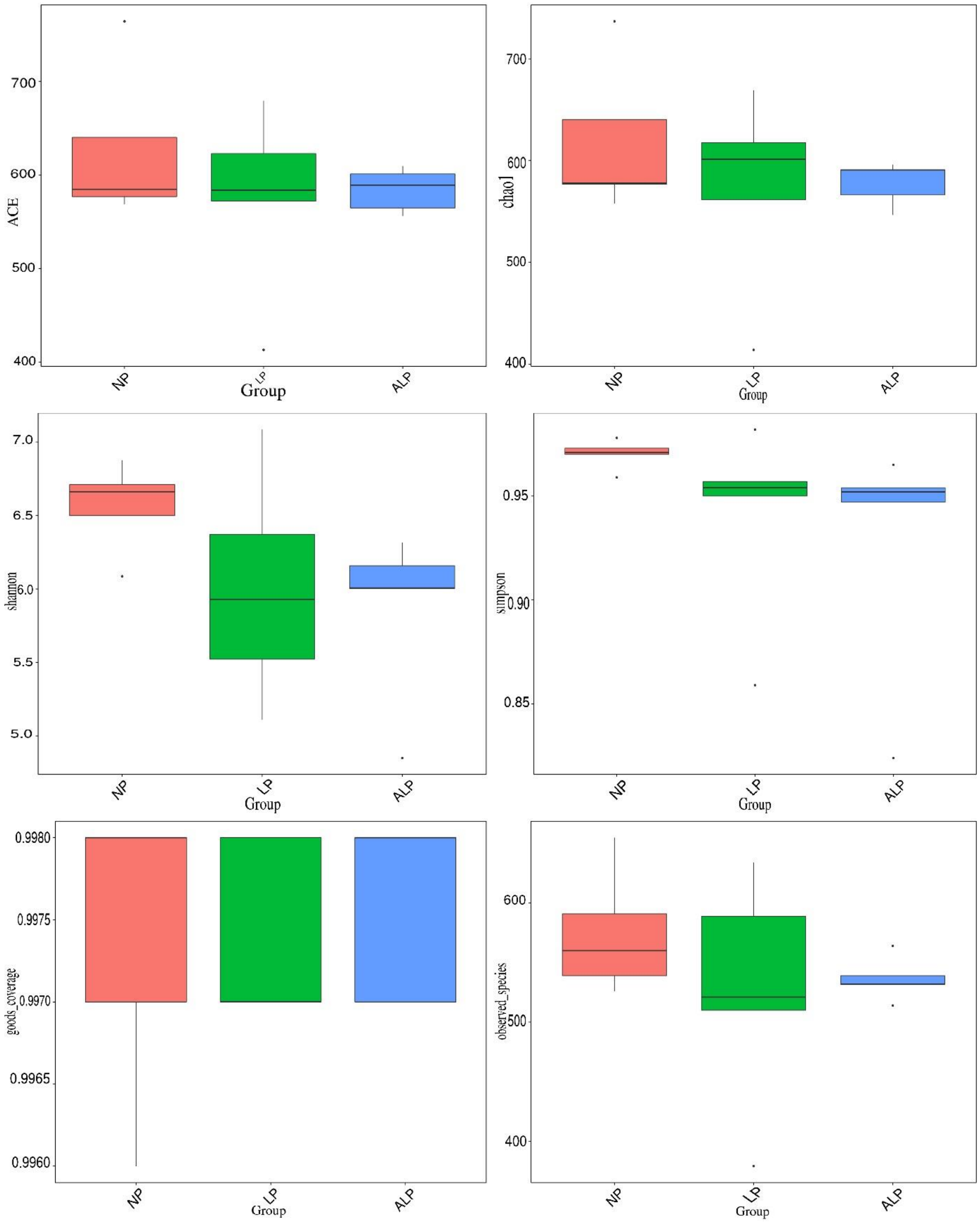

NP, normal protein; LP, low protein; ALP, AKG plus low protein

Supplementary Figure S2.

Comparison of *Oribacterium*, *Phascolarctobacterium* and Lachnospiraceae in cecum among dietary treatments, using real-time PCR analysis. NP, normal protein; LP, low protein; ALP, AKG plus low protein. For each bacterial group, bars not labeled with same letters indicate values are significantly different at $P<0.05$.

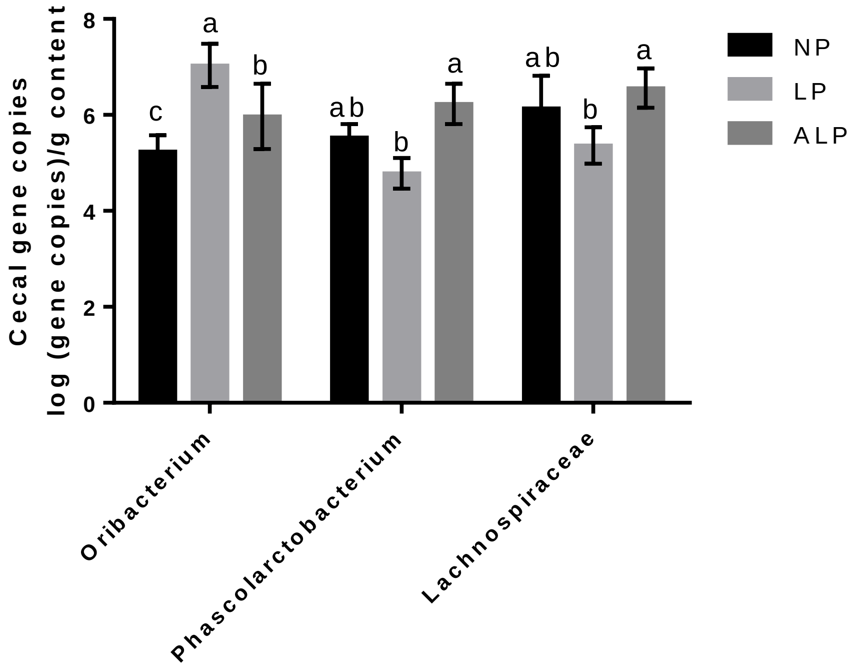

Supplementary Methods

DNA Extraction and PCR Amplification.

Total genomic DNA was isolated from the samples of cecal digesta by using QIAamp DNA Stool Mini Kits according to the manufacturer's instructions. The concentration of the extracted DNA was determined with the NanoDrop-1000 Spectrophotometer (NanoDrop Technologies Inc., Wilmington, DE, USA), DNA purity was monitored on 1% agarose gels. According to the concentration, DNA was diluted to 1ng/μL using sterile water, and was stored at -80℃ before further analysis.

Sequencing was performed at Novogene Bioinformatics Technology Co. Ltd., Beijing, China. The PCR amplifications were conducted with the barcoded primer pair 341f/806r set, which amplifies the V3–V4 fragments of the 16S rDNA gene (341F:CCTAYGGGRBGCASCAG, 806R: GGACTACNNGGGTATCTAAT) (Muyzer et al., 2013; Caporaso et al., 2011). PCR reactions were performed in a volume of 30 μL containing 12 μL sterile water, 1.0 μL DNA template, 1.0 μL of each primer, and 15 μL 2× Phusion Master Mix (New England Biolabs, USA). The PCR cycle conditions were as follows: initial denaturation at 98 ℃ for 1 min, followed by 30 cycles at 98 ℃ for 10 s, 50 ℃ for 30 s, and 72 ℃ for 30 s, and a final extension step at 72 ℃ for 5 min. Resulting amplicons were confirmed on 2% agarose gels containing ethidium bromide..

All amplicons were in the size range of 400–450 bp, and were purified using a GeneJET Gel Extraction Kit (Thermo Fisher Scientific, Carlsbad, CA, USA). Following quantitation, equal concentrations of the purified amplicons were combined into a single tube. Sequencing libraries were generated using a NEB Next Ultra DNA Library Prep Kit for Illumina (New England Biolabs, Ipswich, MA, USA) following manufacturer’s recommendations, and index codes were added. The library quality was assessed on a Qubit @ 2.0 Fluorometer (Thermo Fisher Scientific, Carlsbad, CA, USA) and Agilent Bioanalyzer 2100 system. At last, the library was sequenced on an IlluminaHiSeq2500 platform and 250 bp paired-end.

reads were generated.

Bioinformatics analysis

Paired-end reads was assigned to samples based on their unique barcode and truncated by cutting off the barcode and primer sequence. Paired-end reads were merged using FLASH (V1.2.7,http://ccb.jhu.edu/software/FLASH/) [1],a very fast and accurate analysis tool, which was designed to merge paired-end reads when at least some of the reads overlap the read generated from the opposite end of the same DNA fragment, and the splicing sequences were called raw tags. The tags were compared with the reference database (Gold database, http://drive5.com/uchime/uchime_download.html)using UCHIME algorithm (UCHIME Algorithm, http://www.drive5.com/usearch/manual/uchime_algo.html) [4] to detect chimera sequences, and then the chimera sequences were removed [5]. Then the Effective Tags finally obtained. Sequences analysis were performed by Uparse software (Uparse v7.0.1001，http://drive5.com/uparse/) [6]. Sequences with ≥97% similarity were assigned to the same OTUs. Representative sequence for each OTU was screened for further annotation. For each representative sequence, the GreenGene Database 3(http://greengenes.lbl.gov) [7] was used based on RDP classifier(Version 2.2, http://sourceforge.net/projects/rdp-classifier/)[8]algorithmto annotate taxonomic information.

In order to study phylogenetic relationship of different OTUs, and the difference of the dominant species in different samples(groups), multiple sequence alignment were conducted using the MUSCLE software (Version 3.8.31， http://www.drive5.com/muscle/)[9]. OTUs abundance information were normalized using a standard of sequence number corresponding to the sample with the least sequences.

Alpha diversity is applied in analyzing complexity of species diversity for a sample through 6 indices, including Observed-species, Chao1, Shannon, Simpson, ACE, Good-coverage. All this indices in our samples were calculated with QIIME (Version 1.7.0) and displayed with R software (Version 2.15.3). Beta diversity analysis was used to evaluate differences of samples in species complexity, Beta diversity on both weighted and unweighted unifrac were calculated by QIIME software (Version 1.7.0). Unweighted Pair-group Method with Arithmetic Means (UPGMA) Clustering was

performed as a type of hierarchical clustering method to interpret the distance matrix using average linkage and was conducted by QIIME software (Version 1.7.0).

Real-time polymerase chain reaction analysis of *Oribacterium, Phascolarctobacterium* and Lachnospiraceae.

Total bacterial DNAs were extracted from the contents of each intestinal sample (0.2 g) according to a previously described protocol (Kraler et al., 2016)[12], using a commercially available QIAamp DNA Stool Mini Kit (Qiagen, Hilden, Germany). Those extracts were stored at –80 ℃. They were then quantified on a Nanodrop 2000 Spectrophotometer (Thermo Scientific, Courtaboeuf, France) before the results were adjusted to a concentration of 10 ng μL^{-1}. Methods based on 16S rRNA were used to assess the abundances of *Oribacterium, Phascolarctobacterium* and Lachnospiraceae, as described previously (Kong et al., 2014; Fleury et al., 2016)[13-14] . All PCR primers used in this study are listed in Supplementary table S3.

The qPCR protocol for assaying *Lachnospiraceae* included 0.3 μM of each primer and 0.1 μM of probe, while those reactions for *Oribacterium* and *Phascolarctobacterium* used a concentration of 0.4 μM of each primer. The amplification program entailed 95 ℃ for 30 s; followed by 40 cycles of 95 ℃ for 5 s and 60 ℃ for 30 s; and then a final melting-curve for SYBR Green tests. The melting curve analysis and size-determination of amplificates on agarose gels verified that the target fragments had been amplified. Standard curves were generated as described by Qi et al. (2011) [15]. For each sample and each bacterial group, results were expressed in log10 copies of 16S rRNA genes per g of intestinal content material (Metzlerzebeli et al., 2015)[16].

Reference

[1] Magoč T, Salzberg S L. FLASH: fast length adjustment of short reads to improve genome assemblies. Bioinformatics 27.21 (2011): 2957-2963.

[2] Bokulich, Nicholas A., et al. Quality-filtering vastly improves diversity estimates

from Illuminaamplicon sequencing. Nature methods 10.1 (2013): 57-59.

[3] Caporaso, J. Gregory, et al. QIIME allows analysis of high-throughput community sequencing data. Nature methods 7.5 (2010): 335-336.

[4] Edgar, Robert C., et al. UCHIME improves sensitivity and speed of chimera detection. Bioinformatics 27.16 (2011): 2194-2200.

[5] Haas, Brian J., et al. Chimeric 16S rRNA sequence formation and detection in Sanger and 454-pyrosequenced PCR amplicons.Genome research 21.3 (2011): 494-504.

[6] Edgar, Robert C. UPARSE: highly accurate OTU sequences from microbial amplicon reads. Nature methods 10.10 (2013): 996-998.

[7] DeSantis, Todd Z., et al. Greengenes, a chimera-checked 16S rRNA gene database and workbench compatible with ARB. Applied and environmental microbiology 72.7 (2006): 5069-5072.

[8] Wang, Qiong, et al. Naive Bayesian classifier for rapid assignment of rRNA sequences into the new bacterial taxonomy. Applied and environmental microbiology 73.16 (2007): 5261-5267.

[9] Edgar R C. MUSCLE: multiple sequence alignment with high accuracy and high throughput. Nucleic acids research32.5 (2004): 1792-1797.

[10] Wu F, Guo X, Zhang J, et al. Phascolarctobacterium faecium abundant colonization in human gastrointestinal tract[J]. Experimental & Therapeutic Medicine, 2017, 14(4):3122.

[11] Newton R J, Vandewalle J L, Borchardt M A, et al. Lachnospiraceae and Bacteroidales alternative fecal indicators reveal chronic human sewage contamination in an urban harbor[J]. Applied & Environmental Microbiology, 2011, 77(19):6972-6981.

[12] Kraler, M., Ghanbari, M., Domig, K.J., Schedle, K., Kneifel, W. (2016). The intestinal microbiota of piglets fed with wheat bran variants as characterised by 16S rRNA next-generation amplicon sequencing. Arch. Anim. Nutr. 70, 173-189.

[13] Kong, X.F., Zhou, X.L., Lian, G.Q., Blachier, F., Liu, G., Tan, B.E., et al. (2014).

Dietary supplementation with chitooligosaccharides alters gut microbiota and modifies intestinal luminal metabolites in weaned Huanjiang mini-piglets. Livest. Sci. 160, 97-101.

[14] Fleury, M.A., Jouy, E., Eono, F., Cariolet, R., Couet, W., Gobin, P., et al. (2016). Impact of two different colistin dosing strategies on healthy piglet fecal microbiota. Res. Vet. Sci. 107, 152-160.

[15] Qi, H., Xiang, Z., Han, G., Yu, B., Huang, Z., Chen, D. (2011). Effects of different dietary protein sources on cecal microflora in rats. Afr. J. Biotechnol. 10, 3704-3708. doi: 10.5897/AJB10.2677.

[16] Metzlerzebeli, B.U., Schmitzesser, S., Mann, E., Grüll, D., Molnar, T., Zebeli, Q. (2015). Adaptation of the cecal bacterial microbiome of growing pigs in response to resistant starch type 4. Appl. Environ. Microbiol. 81, 8489-8499. doi: 10.1128/AEM.02756-15.
